# Energy-synchronized X-ray absorption spectroscopy photoemission electron microscopy at Shanghai Synchrotron Radiation Facility (SSRF) for materials science

**DOI:** 10.1107/S1600577525007519

**Published:** 2025-09-10

**Authors:** Junqin Li, Guanhua Zhang, Julong Sun, Zilong Zhao, Ying Zou, Zhenhua Chen, Fangyuan Zhu, Yaobo Huang, Yong Wang, Zefeng Ren, Renzhong Tai, Xueming Yang

**Affiliations:** a Shanghai Synchrotron Radiation Facility, Shanghai Advanced Research Institute Chinese Academy of Sciences Shanghai201204 People’s Republic of China; b State Key Laboratory of Chemical Reaction Dynamics, Dalian Institute of Chemical Physics Chinese Academy of Sciences Dalian116023 People’s Republic of China; ESRF – The European Synchrotron, France

**Keywords:** micro-zone XAS, XAS-PEEM, XMCD/XMLD, energy-resolved PEEM imaging system, GAP-MONO linkage

## Abstract

This study develops an integrated X-ray absorption spectroscopy (XAS) photoemission electron microscopy (PEEM) platform on beamline BL09U at the Shanghai Synchrotron Radiation Facility (SSRF), enabling nanoscale characterization of complex materials through energy-resolved imaging and local-area XAS. By using the wide range of energy tunability, full access to different polarizations and PEEM’s surface sensitivity, we have established a gap–monochromator control system under the *EPICS* framework to synchronize the elliptically polarized undulator (EPU) gap and monochromator energy dynamically, optimizing photon flux stability for absorption fine structure analysis. Combining X-ray magnetic circular dichroism (XMCD) and X-ray magnetic linear dichroism (XMLD) with PEEM and local-area XAS, this platform achieves concurrent mapping of electronic structures and magnetic domains in ferromagnetic nano-patterns, as demonstrated through our studies of Ni_80_Fe_20_ Permalloy using this system. The dual-modal approach bridges synchrotron radiation technology and surface science, offering nanometre-scale spatial resolution in XAS with magnetic domain sensitivity through linearly and circularly polarized X-ray excitation, providing researchers with advanced tools for functional materials analysis through synergistic XAS-PEEM techniques and dynamic control systems.

## Abstract 2

This study develops the energy-resolved photoemission electron microscopy imaging and micro-zone X-ray absorption spectroscopy (XAS) method. By integrating X-ray magnetic circular dichroism, X-ray magnetic linear dichroism and micro-zone XAS, we have achieved spatially resolved electronic structure mapping and magnetic property analysis at the nanoscale level. We have also developed a gap–monochromator linkage control system to optimize photon flux and its stability.

## 1. Introduction

Beamline BL09U at the Shanghai Synchrotron Radiation Facility (SSRF) is a soft X-ray beamline boasting a broad energy range and exceptionally high energy resolution (Xue *et al.*, 2014 ▸). Its energy spectrum spans from 20 eV to 2000 eV, with an energy resolving power *E*/Δ*E* reaching up to 35000 at 867 eV. At present, the beamline offers three experimental end-stations for users: angle-resolved photoemission spectroscopy (ARPES), photoemission electron microscopy (PEEM) and resonant inelastic X-ray scattering (RIXS).

The PEEM technique operates based on the photoelectric effect (Li *et al.*, 2021 ▸; Guo, 2010 ▸), where sample surfaces are excited by light to emit electrons that are directly imaged through electron lenses, enabling full-field nanoscale imaging. With spatial resolution on the nanometre scale, PEEM excels in resolving lateral heterogeneities and surface nano­structures. While conventional UV/laser sources have historical utility, third-generation synchrotron radiation has revolutionized PEEM capabilities through extreme brightness, high collimation, energy tunability and polarization control. These advantages have driven PEEM’s widespread adoption across multidisciplinary fields (Locatelli & Bauer, 2008 ▸) including life sciences, materials science, thin-film growth, chemical reactions, catalysis *etc*.

X-ray absorption spectroscopy (XAS) is a powerful analytical technique for probing the local electronic structure and chemical state of materials (Stohr, 1992 ▸; Koningsberger & Prins, 1988 ▸), providing critical insights into valence states and electronic environments that form the basis for understanding material properties. The technique operates by exposing materials to highly monochromatic X-ray beams, which generate multiple detectable signals through absorption, reflection or transmission phenomena. Specifically, transmitted or reflected X-rays produce absorption spectra, while excited Auger electrons and fluorescence photons enable surface-sensitive chemical analysis and depth profiling of electronic structures, respectively. This multi-signal capability allows concurrent characterization of elemental composition, surface chemistry and bulk electronic properties. Its versatility has established it as an indispensable tool across disciplines, particularly in materials science (*e.g.* catalyst characterization), surface chemistry (*e.g.* interface reaction monitoring), nanotechnology (*e.g.* quantum dot electronic mapping) *etc*.

In XAS research, X-rays in the soft X-ray range play an important role in the near-edge absorption spectrum, primarily corresponding to the *K* edges of carbon and oxygen and the *L*/*M*/*N* edges of many transition metals. These characteristic energy levels enable distinct analytical advantages: carbon/oxygen edges provide novel insights into organic and carbon-based materials through valence-state mapping, while transition metal edges serve as fundamental probes for studying chemical composition and magnetic properties in metals and metal oxides. The complementary nature of these spectral regions allows researchers to investigate simultaneously both surface chemistry and bulk electronic structures in complex materials.

Traditional XAS techniques are inherently limited in resolving microscopic features due to their macroscopic measurement scale. X-ray PEEM addresses this constraint by integrating high spatial resolution with elemental sensitivity, enabling spatially resolved XAS analysis on the micro- to nanoscale. This capability arises from detecting photon energy-dependent variations in electron emission, which directly reflect element-specific absorption characteristics. The versatility of PEEM is further amplified through synergistic combinations with complementary techniques. By coupling with XAS-derived methods, including X-ray magnetic circular dichroism (XMCD) and X-ray magnetic linear dichroism (XMLD), PEEM has emerged as a powerful tool for mapping chemical states, ferromagnetic/antiferromagnetic domains and electronic structures with nanoscale precision. In chemical imaging, it has elucidated:

(i) Quantum confinement effects. Oxidation rate modulation in Mg/W(110) thin films revealed a linear correlation between the Fermi-level density of states (DOS) and surface reactivity (Aballe *et al.*, 2004 ▸).

(ii) Catalytic dynamics. Reaction-driven Au–O phase separation on Rh(110) surfaces demonstrated nonlinear adsorbate reorganization under oscillating conditions (Locatelli *et al.*, 2005 ▸).

In magnetic studies, XMCD-PEEM has uncovered:

(iii) Phase competition. Thickness-dependent ferro­magnetic/paramagnetic stripe domains in MnAs/GaAs heterostructures (Zdyb *et al.*, 2005 ▸).

(iv) Interface spin engineering. Uncompensated spin distributions at Co/NiO interfaces have been found to govern exchange bias effects (Scholl *et al.*, 2004 ▸).

Beyond conventional materials, XANES-PEEM has expanded into biomineralization research, revealing:

(v) Biologically templated crystallization. Microbe-induced growth of FeOOH pseudo-single crystals via polysaccharide templating (Chan *et al.*, 2004 ▸).

(vi) Hierarchical architectures. Organic matrix-directed aragonite tablet alignment in nacre, explaining its fracture-resistant properties (Metzler *et al.*, 2007 ▸).

In this work, we have developed an integrated XAS-PEEM platform. A data acquisition system has been integrated, which links and integrates the PEEM imaging system with the monochromator energy controlled by *EPICS*, achieving energy-resolved PEEM imaging. This system also dynamically synchronizes the elliptically polarized undulator (EPU) gap and monochromator energy, which optimizes the stability of the photon flux for absorption fine structure analysis. By integrating XMCD, XMLD, PEEM and local-area XAS, the platform can simultaneously map the electronic structures and magnetic domains in ferromagnetic nano-patterns.

## 2. Description of the experimental system

**Figure 1:**
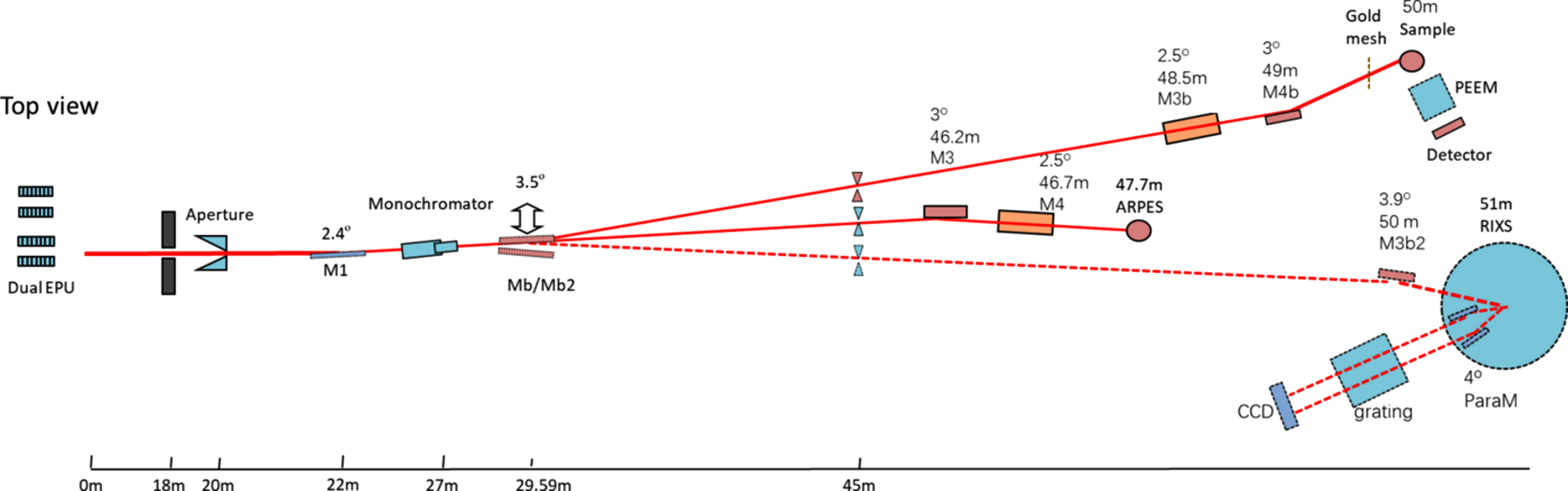
The X-ray beamline layout.

The experimental system encompasses an X-ray source with adjustable energy and polarization, a spectroscopic photoemission and low-energy electron microscopy system, and an advanced data acquisition system. The beamline layout is shown in Fig. 1 ▸. After exiting the EPU, the X-rays are dispersed by a monochromator to select the target energy, then focused by a Kirkpatrick–Baez (KB) mirror system (M3b and M4b) before reaching the experimental station.

### 2.1. Tunable X-ray source with adjustable energy and polarization

The source of BL09U utilizes dual APPLE II-type elliptical polarization undulators (Sasaki, 1994 ▸; Lidia & Carr, 1994 ▸), enabling seamless energy regime switching between 20–200 eV (low-energy EPU, LEID) and 200–2000 eV (high-energy EPU, HEID) while maintaining stable electron beam orbits. X-ray energy and polarization states are precisely tunable via gap/shift parameter adjustments (Zou *et al.*, 2019 ▸). In order to meet the diverse experimental requirements for energy resolution while achieving an exceptional energy resolving power *E*/Δ*E* up to 35000 at 867 eV, the system incorporates a variable-included-angle plane grating monochromator (Reininger, 2011 ▸) with four gratings: low-energy (400 l mm^−1^, LEG), medium-energy (800 l mm^−1^, MEG), high-energy (1200 l mm^−1^, HEG) and very-high-resolution (3600 l mm^−1^, VEG). This multi-grating configuration achieves a wide X-ray beam energy across 20–2000 eV, supporting diverse experimental demands while enabling precise characterization of material electronic structures and chemical states through synchrotron-based X-ray spectroscopy.

### 2.2. Photoemission electron microscope

**Figure 2:**
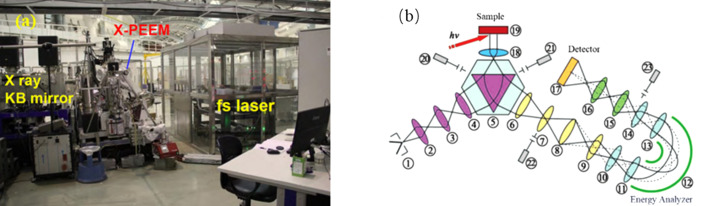
(*a*) Photograph of the X-PEEM end-station at the SSRF. (*b*) Schematic diagram of the electron optics in PEEM showing components of the SPELEEM system (Guo, 2010 ▸). (1) Electron gun. (2) Focusing electromagnetic lens 1. (3) Focusing electromagnetic lens 2. (4) Focusing electromagnetic lens 3. (5) Electron beam splitter. (6) Transfer electromagnetic lens. (7) Field electromagnetic lens. (8) Intermediate electromagnetic lens. (9) Projective electromagnetic lens 1. (10) Decelerating lens. (11) Imaging lens 1. (12) Electron energy analyzer. (13) Imaging lens 2. (14) Accelerating lens. (15) Projective electromagnetic lens 2. (16) Projective electromagnetic lens 3. (17) MCP and fluorescent screen. (18) Objective lens. (19) Sample. (20) Illumination aperture. (21) Selected area aperture. (22) Contrast aperture. (23) Energy analyzer slit. The solid line represents imaging mode, while the dotted line represents electron diffraction mode.

The PEEM station at the SSRF [shown in Fig. 2 ▸(*a*)] incorporates the SPELEEM III system from Elmitec, Germany (Bauer *et al.*, 1997 ▸; Bauer, 2001 ▸), enabling comprehensive materials science research through integrated imaging, diffraction and photoelectron spectroscopy capabilities. The system operates based on imaging with photo-excited electrons or surface-reflected electrons. As shown in Fig. 2 ▸(*b*), when samples are irradiated by light sources (femtosecond lasers, UV or synchrotron radiation) or electron beams, emitted/back reflected electrons are collected by objective lenses, transferred through electron optical elements and projected onto detectors to form magnified images (Li *et al.*, 2019 ▸). This process simultaneously achieves high-resolution surface morphology imaging and dynamic electron emission analysis under various excitation conditions.

In addition, the instrument is equipped with a preparation chamber that offers sample processing functionalities, including potassium (K) and caesium (Cs) source dosing, argon (Ar) ion sputtering, and gas introduction. These features enable *in situ* sample preparation for various characterization needs, ensuring comprehensive experimental flexibility. The main chamber of the SPELEEM instrument is equipped to provide MBE (molecular beam epitaxy), EBM (electron beam melting) and controlled atmospheric environ­ments, enabling real-time *in situ* growth observation. These capabilities are available for user operations.

The experimental station extends beyond standard low-energy electron microscopy (LEEM) with electron-gun and mercury-lamp PEEM capabilities by incorporating femto­second laser and synchrotron X-ray sources for advanced PEEM imaging (Li *et al.*, 2019 ▸). Specifically in X-PEEM mode, X-rays impinge on samples at a 16° incidence angle on downward-facing surfaces – which can be azimuthally rotated – enabling high-resolution mapping of surface structures and composition distributions through element-specific imaging via core-level photoemission or photon energy tuning to absorption edges. Beam conditioning is achieved through a vertical-defining two-blade exit slit; at 525 eV photon energy with 60 µm vertical slit setting, this yields a light spot full width at half-maximum (FWHM) of 115 µm (H) × 13 µm (V). Detection employs a microchannel plate (MCP) with a phosphor screen and PCO.1600X CCD camera, featuring Elmitec’s safety mechanism against overexposure that automatically elevates the start voltage when the camera intensity exceeds a threshold to protect the MCP from electron flux damage. The SPELEEM instrument delivers 17 nm spatial resolution performance.

In this work, we expanded the experimental capabilities of the X-PEEM station by developing energy-resolved PEEM imaging, local-area XAS and XMCD/XMLD techniques. Exploiting synchrotron radiation’s tunable energy and polarization properties, we have established advanced method­ologies for correlated electron–magnetic–structural characterization, significantly enhancing the experimental station’s versatility and research impact in materials science.

### 2.3. Implementation of energy-resolved PEEM imaging and micro-zone XAS techniques

First, synchrotron radiation X-rays are focused onto the sample surface to generate photoelectrons. For XAS measurement, secondary electrons, the yield of which is proportional to the absorption of the X-rays, are selected to produce PEEM images. Photon energy-resolved PEEM imaging is achieved by systematically adjusting the monochromator’s reflecting angle to select specific X-ray wavelengths, and acquiring secondary-electron PEEM images at each discrete energy value.

The SSRF beamline control system utilizes the *Experimental Physics and Industrial Control System* (*EPICS*) (Kraimer *et al.*, 2018 ▸; see also https://epics.anl.gov/). To enhance experimental efficiency near elemental absorption edges, we have developed a *LabView*-based automated data acquisition program that integrates with *EPICS* through a dedicated interface library, enabling concurrent control of the EPU, monochromator and camera systems. This program automates the workflow by configuring camera exposure time and image-size parameters, initializing monochromator energy positions, and sequentially capturing single-energy images across the target energy range. Meanwhile, the photocurrent produced at the gold mesh by each energy X-ray before entering PEEM is also recorded, which represents the photon flux. By moving the monochromator stepwise and repeating the image acquisition, the system rapidly generates a comprehensive PEEM image series near the absorption edge of the element under study.

This integrated approach not only streamlines energy-resolved imaging but also ensures precision in multi-device synchronization, significantly improving experimental throughput. The resulting high-resolution image dataset forms a robust foundation for advanced analysis of electronic structures and chemical states in materials science research.

The energy-resolved PEEM imaging system not only acquires high-resolution images but also synchronously collects secondary electron yield data from the interrogated regions. The principle is as follows. After X-rays have interacted with the sample to generate secondary electrons, these electrons are transmitted to the MCP. The multiplied electrons from the MCP strike the fluorescent screen, generating fluorescence, which is then recorded by the CCD. The count of fluorescent photons recorded by the CCD is directly proportional to the number of secondary electrons. It also records the photocurrent on the gold mesh upstream of the experimental station, which represents photon flux information for the experimental station. By analyzing the energy-dependent variation in electron yield, we can reconstruct the sample’s absorption spectrum, revealing its electronic structure response to different excitation energies. Microscopic areas or structures of specific interest can be precisely targeted from the XAS-PEEM images, especially at the absorption edge energies where intensity contrast is usually observed. Spatially resolved local-area absorption spectra can be extracted from any surface region of interest with a size down to dozens of nanometres.

This dual functionality – combining spectral characterization and local-area spectroscopic analysis – expands the application of XAS beyond traditional bulk material studies to complex nanostructured systems. The automated data processing pipeline, which integrates real-time yield data acquisition with spectral reconstruction algorithms, provides a comprehensive toolkit for investigating material properties such as optical characteristics, chemical states and magnetic domain distributions in advanced research fields including surface chemistry, nanotechnology and condensed matter physics. The typical optimal energy resolution for XAS measurements is 0.15 eV.

### 2.4. XMCD/XMLD for studying the microstructures of magnetic materials

Based on the above techniques, the XMCD/XMLD method was established and tested. Both dichroism methods are used to probe the electronic configuration modifications in magnetic systems, manifested through energy-dependent intensity variations in the near-edge X-ray absorption fine structure. The observed spectral changes arise from the interplay between the material’s magnetic moments and the incident X-ray polarization, specifically depending on the magnetic moment’s magnitude and orientation (Alders *et al.*, 1998 ▸; Schütz *et al.*, 1987 ▸).

**Figure 3:**
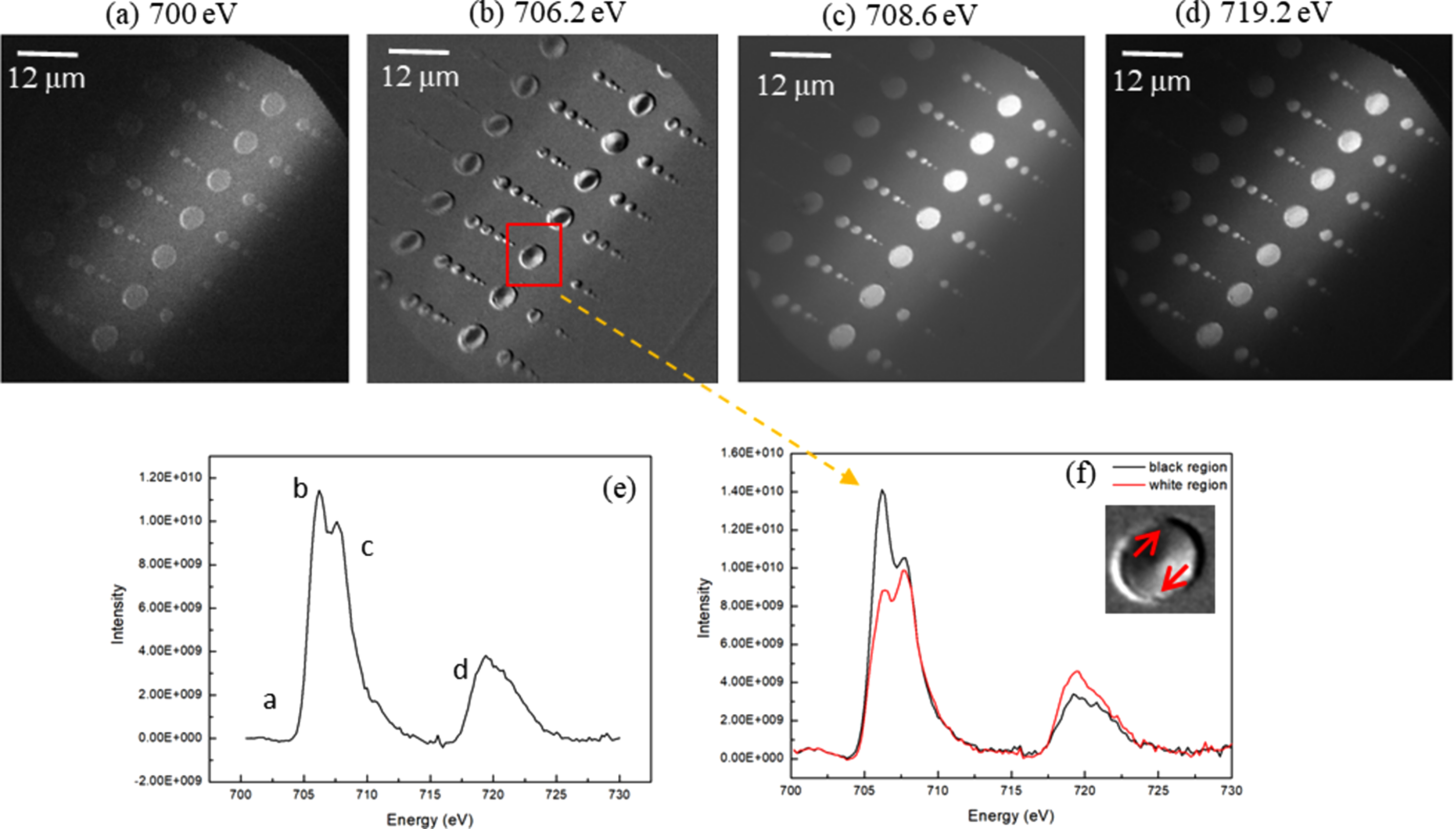
PEEM images taken at energies of (*a*) 700 eV, (*b*) 706.2 eV, (*c*) 708.6 eV and (*d*) 719.2 eV. (*e*) The integrated absorption spectrum for the whole imaged region. The energy points labeled successively as a, b, c and d correspond to panels (*a*), (*b*), (*c*) and (*d*), respectively. (*f*) Absorption spectra for two selected areas with different contrast in the disk enclosed by the red box in panel (*b*).

Experimentally, we employed Ni_80_Fe_20_ Permalloy nanopatterns prepared on Si substrates through magnetron sputtering for PEEM studies. The imaging field contained arrays of Ni_80_Fe_20_ disks exhibiting vortex magnetic domain structures, with diameters varying between 300 nm and 6000 nm and uniform thickness (∼50 nm). To elucidate the electronic and magnetic characteristics of these nanostructures, we conducted energy-resolved PEEM measurements near the Fe *L* edge (700–730 eV) using circularly polarized X-rays with a step size of 0.2 eV. For each energy, we determined the signal intensity across the entire patterned area, thereby constructing the element-specific absorption spectrum as illustrated in Fig. 3 ▸(*e*). This approach simultaneously provides both morphological information about the magnetic domains and quantitative insights into the material’s electronic state evolution. The other experimental details for the images are: exposure time 400 ms, averaging over ten frames, ring current 200 mA and flux on gold grid 3 × 10^−8^ A.

Figs. 3 ▸(*a*)–3 ▸(*d*) display the energy-dependent PEEM imaging results for Ni_80_Fe_20_ nanodisks at 700 eV, 706.2 eV, 708.6 eV and 719.2 eV, respectively. These images reveal intensity contrasts corresponding to variations in the secondary electron yield across the magnetic domains. Notably, the Fe *L*_3_ edge (706.2 eV) and *L*_2_ edge (719.2 eV) images [Figs. 3 ▸(*b*) and 3 ▸(*d*)] exhibit pronounced vortex-like magnetic domain structures characterized by distinct bright/dark contrast regions. The enhanced intensity modulation at 706.2 eV [Fig. 3 ▸(*b*)] facilitates detailed morphological analysis of these magnetic vortices.

To investigate the electronic origins of the observed contrast variations on a representative vortex from Fig. 3 ▸(*b*), we extracted the XAS spectra of two regions with contrasting intensities: one exhibiting bright emission (high-intensity region) and the other showing dark emission (low-intensity region). As shown in Fig. 3 ▸(*f*), the spectral data demonstrate significant differences in the Fe 3*d* electronic states between the two micro-regions, directly correlating with their distinct magnetic moment configurations.

By analyzing the circularly polarized X-ray induced transitions at the Fe *L*_3_ (2*p*_3/2_ → 3*d*) and *L*_2_ (2*p*_1/2_ → 3*d*) edges, we can establish a direct relationship between the electronic structure evolution and the spatial distribution of magnetic domains. The energy-selective imaging approach not only provides morphological characterization of the vortex structures but also enables quantitative analysis of the spin-dependent electron distribution within the nano-patterned material.

In addition to XMCD, XMLD can also be performed. as described by Xu *et al.* (2019 ▸). This work demonstrates that antiferromagnetic domains are present in NiO(001) thin films grown on MgO(001) and confirms the AFM origin of the optical contrast via XMLD-PEEM measurements.

### 2.5. GAP-MONO coupled XAS method

Optimization of light intensity parameters is essential for X-ray spectroscopic techniques: a high photon flux enhances the signal strength for improved spatial resolution in XMCD/ XMLD imaging and chemical-state mapping, while a stable intensity minimizes spectral distortions in XAS by reducing intensity fluctuations that necessitate post-processing normalization. This study focuses on achieving a simultaneous high signal quality and spectral accuracy in absorption fine structure studies by precisely coordinating the energy-dependent EPU gap adjustment with X-ray monochromator positioning (GAP-MONO linkage), ensuring maximum photon flux at every energy point across the scanning range. The dual requirements for intensity modulation – elevated flux for contrast enhancement versus strict stability for spectral fidelity – are resolved through synchronized control of optical components, establishing a systematic framework for multimodal X-ray data acquisition.

Building on the previously established data acquisition software for the energy-resolved PEEM imaging system, we developed enhanced control interfaces between the experimental station and the *EPICS* framework. This integration allows the system automatically to synchronize the EPU gap with the monochromator energy settings, thereby implementing a GAP-MONO-linked local-area XAS technique. During energy scans, the gap is synchronously adjusted to optimize the luminous flux at each energy point. For soft X-ray elements exhibiting absorption spectra spanning tens of electronvolts, we observed a quasi-linear relationship between the maximum photon flux and the X-ray energy. Based on this empirical observation, we established a linear regression model: Gap = *K* × *E* + *b*, where *K* and *b* are calibration coefficients determined by measuring the gap values at the start/stop energy points before experimentation.

**Figure 4:**
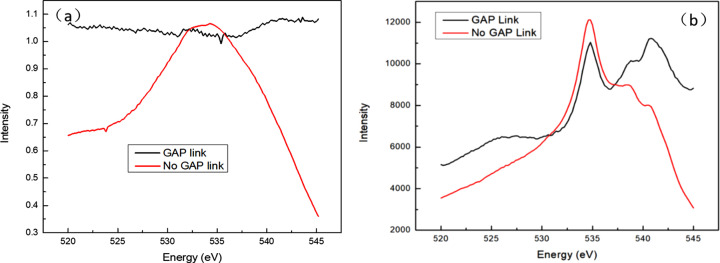
Comparison of (*a*) photon flux and (*b*) X ray absorption spectra for coupled (GAP link) and uncoupled (no GAP link) modes.

Experimental validation demonstrates the effectiveness of this approach through comparative measurements of the oxygen edge spectrum of an SrTiO_3_ sample in coupled versus uncoupled modes (Fig. 4 ▸). Photon flux data, derived from the upstream gold mesh photocurrent, shows significant improvement across the scan range when using the GAP-MONO linkage method. The peak at 542 eV is more prominent when using the GAP-MONO linkage method, while without gap-coordinated adjustment this peak only appears as a small bulge. It can be observed that the peak position information is clearer in the GAP-MONO linkage mode. This optimization not only stabilizes the normalization reference but also enhances the spectral resolution, providing researchers with superior conditions for analyzing fine structural features in absorption spectra.

## 3. Conclusion

In summary, this work establishes a comprehensive experimental tool combining the tunable energy and polarization capabilities of the PEEM endstation on beamline BL09U at the SSRF with advanced energy-resolved PEEM techniques. By integrating XMCD/XMLD and local-area XAS, we have achieved spatially resolved electronic structure mapping and magnetic property analysis at the nanoscale level. This dual-modal approach enables precise identification of optical properties in complex materials, providing powerful tools for frontier research in condensed matter physics and nanotechnology.

In order to meet the needs for stable photon flux control in absorption spectrum fine structure studies, we developed a GAP-MONO linkage that dynamically adjusts the EPU gap with the monochromator energy settings. Through linear regression modeling based on calibration measurements at energy extremes, this innovation achieves real-time optimization of photon flux stability across extended energy ranges. The developed system demonstrates superior performance in resolving fine spectral features, as validated through our Ni_80_Fe_20_ Permalloy nanopattern studies.
